# Severely Attenuated Visual Feedback Processing in Children on the Autism Spectrum

**DOI:** 10.1523/JNEUROSCI.1192-22.2023

**Published:** 2023-03-29

**Authors:** Emily J. Knight, Edward G. Freedman, Evan J. Myers, Alaina S. Berruti, Leona A. Oakes, Cody Zhewei Cao, Sophie Molholm, John J. Foxe

**Affiliations:** ^1^Frederick J. and Marion A. Schindler Cognitive Neurophysiology Laboratory, Del Monte Institute for Neuroscience, Department of Neuroscience, University of Rochester School of Medicine and Dentistry, Rochester, New York 14642; ^2^Development and Behavioral Pediatrics, Golisano Children's Hospital, University of Rochester, Rochester, New York 14642; ^3^Cognitive Neurophysiology Laboratory, Department of Pediatrics and Neuroscience, Albert Einstein College of Medicine, Bronx, New York 10461

**Keywords:** autism spectrum disorder, illusory contours, object recognition, visual evoked potentials, visual feedback

## Abstract

Individuals on the autism spectrum often exhibit atypicality in their sensory perception, but the neural underpinnings of these perceptual differences remain incompletely understood. One proposed mechanism is an imbalance in higher-order feedback re-entrant inputs to early sensory cortices during sensory perception, leading to increased propensity to focus on local object features over global context. We explored this theory by measuring visual evoked potentials during contour integration as considerable work has revealed that these processes are largely driven by feedback inputs from higher-order ventral visual stream regions. We tested the hypothesis that autistic individuals would have attenuated evoked responses to illusory contours compared with neurotypical controls. Electrophysiology was acquired while 29 autistic and 31 neurotypical children (7-17 years old, inclusive of both males and females) passively viewed a random series of Kanizsa figure stimuli, each consisting of four inducers that were aligned either at random rotational angles or such that contour integration would form an illusory square. Autistic children demonstrated attenuated automatic contour integration over lateral occipital regions relative to neurotypical controls. The data are discussed in terms of the role of predictive feedback processes on perception of global stimulus features and the notion that weakened “priors” may play a role in the visual processing anomalies seen in autism.

**SIGNIFICANCE STATEMENT** Children on the autism spectrum differ from typically developing children in many aspects of their processing of sensory stimuli. One proposed mechanism for these differences is an imbalance in higher-order feedback to primary sensory regions, leading to an increased focus on local object features rather than global context. However, systematic investigation of these feedback mechanisms remains limited. Using EEG and a visual illusion paradigm that is highly dependent on intact feedback processing, we demonstrated significant disruptions to visual feedback processing in children with autism. This provides much needed experimental evidence that advances our understanding of the contribution of feedback processing to visual perception in autism spectrum disorder.

## Introduction

Individuals on the autism spectrum demonstrate atypicality in sensory processing across multiple domains. Given that efficient integration of basic sensory information forms a foundation for more complex cognition and social communication, understanding the mechanisms of basic sensory perception in autism spectrum disorder (ASD) has potential cascading implications for downstream functions (Simmons et al., 2009). A proposed unifying explanation for differences observed in processing across sensory domains is that individuals with autism may have altered top-down modulatory feedback to lower-level sensory processing regions (Smith et al., 2015). In complex environments, top-down feedback that takes into account prior experience and contextual information supports efficient sensory processing by helping to anticipate sensory inputs, address ambiguity, and fill in missing information (Pascual-Leone and Walsh, 2001; Juan and Walsh, 2003). In the literature on visual processing in ASD, this has been variously expressed as a key component of several theories, including enhanced processing of local stimulus features (Bertone et al., 2005; Mottron et al., 2006), weakened processing of central coherence (Frith and Happe, 1994; Happe and Booth, 2008), or weakened application of prior knowledge to the processing of incoming sensory data (sometimes referred to as predictive coding deficits or “hypo-priors”) (Pellicano and Burr, 2012) among those with ASD. Such atypicality is thought to arise from anomalous brain connectivity, including impairments in frontal-parietal-occipital (Damarla et al., 2010) and interhemispheric connectivity (Bertone et al., 2005; Vandenbroucke et al., 2008) during visuospatial processing. Indeed, autism-associated differences in interhemispheric and intrahemispheric connectivity have been increasingly highlighted as an important factor underlying neural processing of even very basic visual phenomena, including long-latency flash visual evoked potentials (VEPs), binocular rivalry, and other paradigms interrogating excitatory-inhibitory imbalances during low-level visual processing (Isler et al., 2010; Seymour et al., 2019; Spiegel et al., 2019). These converging notions of possible links between disrupted early visual processing and anomalous connectivity in autism emerging from such a wide variety of fundamental paradigms highlight the widespread potential relevance of empiric characterization of the role of neural feedback in visual processing for this population.

There has been much interest in using visual illusions as paradigms that allow for probing of function in these feedback systems. Visual illusions use unique combinations of stimulus properties that generate erroneous perception and, in doing so, help elucidate the processes by which the brain integrates stimulus features to form a coherent visual percept, a gestalt. A particularly valuable visual illusion to specifically evaluate feedforward and feedback inputs to visual processing is the Kanizsa illusory contour (IC) (Kanizsa, 1987). Kanizsa ICs consist of Pac-Man shaped inducers that can be aligned such that the inducer cut-outs collectively produce the illusion of a simple two-dimensional shape. This is conceptually similar to situations commonly encountered in real world visual perception where a partially obstructed or fragmented view of an object must be mentally “filled in” to produce a complete object image, but the simplicity of Kanizsa figures affords a relatively straightforward means of assaying contour integration processes using electrophysiological measures of the VEP. As a result, there is a substantial body of literature describing specific behavioral and neurophysiologic correlates of IC processing in both animal models and humans with and without developmental disabilities (Murray and Herrmann, 2013).

Evidence from several studies has accumulated to provide a VEP phenotype when a neurotypical (NT) individual is viewing a Kanizsa figure IC compared with the same set of inducers in random orientation such that they do not form an illusory shape, termed a noncontour (NC) stimulus (Sugawara and Morotomi, 1991; Murray et al., 2002; Proverbio and Zani, 2002; Brodeur et al., 2006; Altschuler et al., 2012). IC processing is best described by a two-phase model where the presence and proportion of each phase are dependent on the observer's age and stimulus features, including complexity and location (Murray et al., 2002; Proverbio and Zani, 2002; Seghier and Vuilleumier, 2006; Shpaner et al., 2009; Poscoliero and Girelli, 2018). The first phase is typically seen between 90 and 200 ms following a visual stimulus and occurs as more negative amplitude in the N1/N170 waveform when viewing an IC relative to NC. This phase has been termed the “perceptual phase” or is often referred to as the IC effect. The IC effect is thought to reflect automatic filling in of boundaries (Vuilleumier et al., 2001; Murray et al., 2002; Shpaner et al., 2009) and is present in both NT children and adults (Altschuler et al., 2014). The second phase occurs between 230 and 400 ms following visual stimuli and is characterized by a sustained negativity evoked by IC relative to NC (Murray et al., 2006). This later phase has been termed the “conceptual phase” but is more commonly known as negativity for closure (*N_cl_*). The *N_cl_* is thought to be associated with more effortful processing and is particularly prominent during “perceptual closure” type exercises whereby recurrent processing gradually “fills in” fragmented complex objects until a coherent percept is recognized (Doniger et al., 2000, 2001; Foxe et al., 2005; Sehatpour et al., 2008). However, an analogous effect is notable in NT adults when they are executing tasks that are explicitly dependent on recognition of an IC (Murray et al., 2006), or in lieu of the earlier IC effect when Kanizsa stimuli are presented in the peripheral visual field (Murray et al., 2002).

Both the IC effect and *N_cl_* have been primarily localized to the lateral occipital complex (Murray et al., 2002, 2004; Sehatpour et al., 2006). While there is some evidence for early contour integration in primary visual cortex as well (Grosof et al., 1993; Hirsch et al., 1995; Lesher, 1995; Leventhal et al., 1998; Seghier and Vuilleumier, 2006; Wokke et al., 2013; Chernyshev et al., 2016), when observed this appears to be likely moderated by top-down feedback from higher-order extrastriate regions as well (Grossberg and Raizada, 2000; Ramsden et al., 2001; Halgren et al., 2003; Maertens et al., 2008; Muckli, 2010; Wokke et al., 2013; Kok and de Lange, 2014; Pak et al., 2020; Pang et al., 2021).

While the dynamics of IC processing have been most extensively described in adults, there is evidence that IC perception matures gradually over early childhood. A 2014 study that assayed the NT development of contour integration from age 6 through adulthood observed more widely distributed and protracted processing of IC stimuli among children compared with adults (Altschuler et al., 2014). Thus, development toward an adult-like VEP pattern is preceded by a greater prominence of later stage *N_cl_* processing, comparable to what has been observed in adults during effortful processing while viewing more complex stimuli or lateral IC presentations. Behaviorally, it has also been noted that NT children under 5 years old are unable to discriminate IC from NC stimuli and tend to look more at the inducer elements, whereas by age 7, NT children exhibit highly accurate discrimination of IC from NC stimuli and direct eye gaze toward the center of the illusory shapes (Nayar et al., 2015).

Given the well-characterized nature of IC processing across NT development and the utility of IC stimuli in probing the top-down recurrent feedback processes that are frequently implicated in ASD, it is of substantial interest to compare IC perception between NT and ASD individuals. Unfortunately, previous behavioral studies of IC perception in ASD children and adults have produced mixed results. Many have described no behavioral difference in closed contour integration in ASD relative to NT children or adults (Milne and Scope, 2008; Hadad et al., 2019; Gowen et al., 2020). Yet, other studies have found ASD children exhibited lower performance than NT controls on tasks that required identification of illusory shapes (Soroor et al., 2022), and lower accuracy along with longer reaction time in matching a solid shape with two illusory alternatives in the presence of local interference (Nayar et al., 2017), implying a possible deficit in contour processing. Atypical patterns of eye gaze during tasks dependent on IC processing have also been observed with ASD children demonstrating fewer fixations on the center of the Kanizsa figures (Nayar et al., 2017), suggestive of a reduced emphasis on global stimulus characteristics.

Electrophysiologic investigation of IC processing in ASD has been more limited, but prior work on preschool-aged children suggests differences in the neural dynamics of contour integration between NT and ASD children. In one such study, Stroganova et al. (2007) revealed an inversion of the IC effect in very young male ASD children between the ages of 3 and 6, whereby IC stimuli generated a more positive N1 amplitude than NC stimuli. Additionally, studies of oscillatory dynamics in both NT and ASD children have revealed typical early occipital γ suppression between 40 and 120 ms but alterations in parietal γ activity beginning at ∼100 ms after stimulus onset (Stroganova et al., 2012). Alterations in γ dynamics relative to NT controls have also been detected in a smaller sample, including 6 adolescents with ASD (Brown et al., 2005). Finally, ASD children (age 4-7 years) demonstrated absence of the NT pattern of IC-evoked parietal alpha power augmentation 133-167 ms after stimulus onset, and instead some ASD children showed evidence of earlier IC processing over the occipital cortex suggesting increased recruitment of lower level processing (Stroganova et al., 2011). This is a pattern that has been observed in processing of other types of visual illusions as well; ASD individuals in general seem to have stronger recruitment of early occipital and temporal processing regions than higher-order prefrontal and parietal activation seen in NT when processing visual illusions (Lin et al., 2017). Together, the studies of oscillatory dynamics of contour integration in ASD reveal disturbances in the underlying processing of IC stimuli that may not be completely captured by behavioral paradigms alone. However, the majority of the electrophysiologic work to date has focused on preschool age children, and it remains unclear the extent to which atypicality in IC-evoked potentials observed in ASD represent delayed versus divergent development of contour integration processing in ASD.

Here, we explore this question by probing neural contour integration systems through direct measurement of VEP responses to both centrally and laterally presented Kanizsa figures in ASD and NT school-aged children and adolescents. Given prior work, multiple outcomes were considered possible. (1) Early automatic contour integration, as indexed by the IC effect, could be entirely absent in ASD. Such a result would point to disordered contour integration in ASD. (2) The developmental trajectory could differ, such that children with ASD are reliant on late conceptual phase processing until a later age than NT children. That is, one might predict that early IC effect processes are attenuated and that later perceptual closure (*N_cl_*) processes are enhanced to compensate. (3) Onset of automatic processing could be intact but delayed, which would suggest that children with ASD may exhibit IC processing that is inefficient. (4) It is possible that the IC effect would be intact but that perceptual closure (*N_cl_*) processes would be selectively attenuated. Given that both the IC effect and the *N_cl_* have been shown to depend on feedback processing, this pattern of effects would imply a dissociation between the feedback systems supporting the two components of processing such that rapid feedforward and feedback processing underlying the IC effect remain intact, but there is selective impairment in feedback processes supporting more effortful closure processes.

## Materials and Methods

### Participants

Thirty-one NT and 29 ASD children (age 7-17 years) took part in the study. Participant characteristics are outlined in Table 1. Participants all had normal or corrected-to-normal vision and normal hearing. Exclusion criteria consisted of history of traumatic brain injury, schizophrenia, bipolar disorder or psychosis, history of neurologic disorder, or an identified syndromic cause for ASD (e.g., Down's syndrome, Fragile X, tuberous sclerosis). NT participants endorsed no diagnosis of any developmental disability nor history of special education. Children with ASD who had an additional diagnosis of attention deficit hyperactivity disorder (ADHD) were not excluded from the study given the high rates of inattention and hyperactivity in this population. However, parents of ASD participants on stimulant medication were asked to refrain from administering the stimulant medication on the day of electrophysiological testing (24 h washout).

**Table 1.** Participant characteristics*^a^*

	NT (*n* = 31)	ASD (*n* = 29)	Significance (*p*)
Mean age (SD)	12 (3)	13 (2)	0.066
Mean Full-Scale IQ (SD)	112 (9.9)	102 (15.9)	0.007
Mean ADOS-2 Comparison Score (SD)	NA	8 (1.5)	NA
Mean Social Responsiveness Scale Total T Score (SD)	46 (7.8)	69 (10.3)	<0.001
Percentage male	32.3%	89.7%	<0.001

*^a^*IQ data not collected for *n* = 9 participants who were lost to follow-up. Missing values excluded from mean IQ calculation. SD: standard deviation.

All experimental procedures were approved by the institutional review boards of the University of Rochester Medical Center and Albert Einstein College of Medicine. Data were collected at both of these sites with cross-site validation of the paradigm and procedures to ensure consistency of data collection. Written informed consent was obtained from the parent or guardian, and children provided developmentally appropriate assent. Participants were modestly compensated for their time in the laboratory. All individuals completed a comprehensive phenotyping battery consisting of IQ testing with the Wechsler Abbreviated Scale of Intelligence, Ed 2 or Wechsler Intelligence Scale for Children, Ed 5; the Swanson, Nolan and Pelham Questionnaire (SNAP-IV) for assessment of ADHD symptoms; and the Social Responsiveness Scale, Ed 2 (SRS-2). ASD diagnoses were validated by administration of the Autism Diagnostic Observation Schedule, Ed 2 (ADOS-2).

### Experimental task

**Figure 1.**
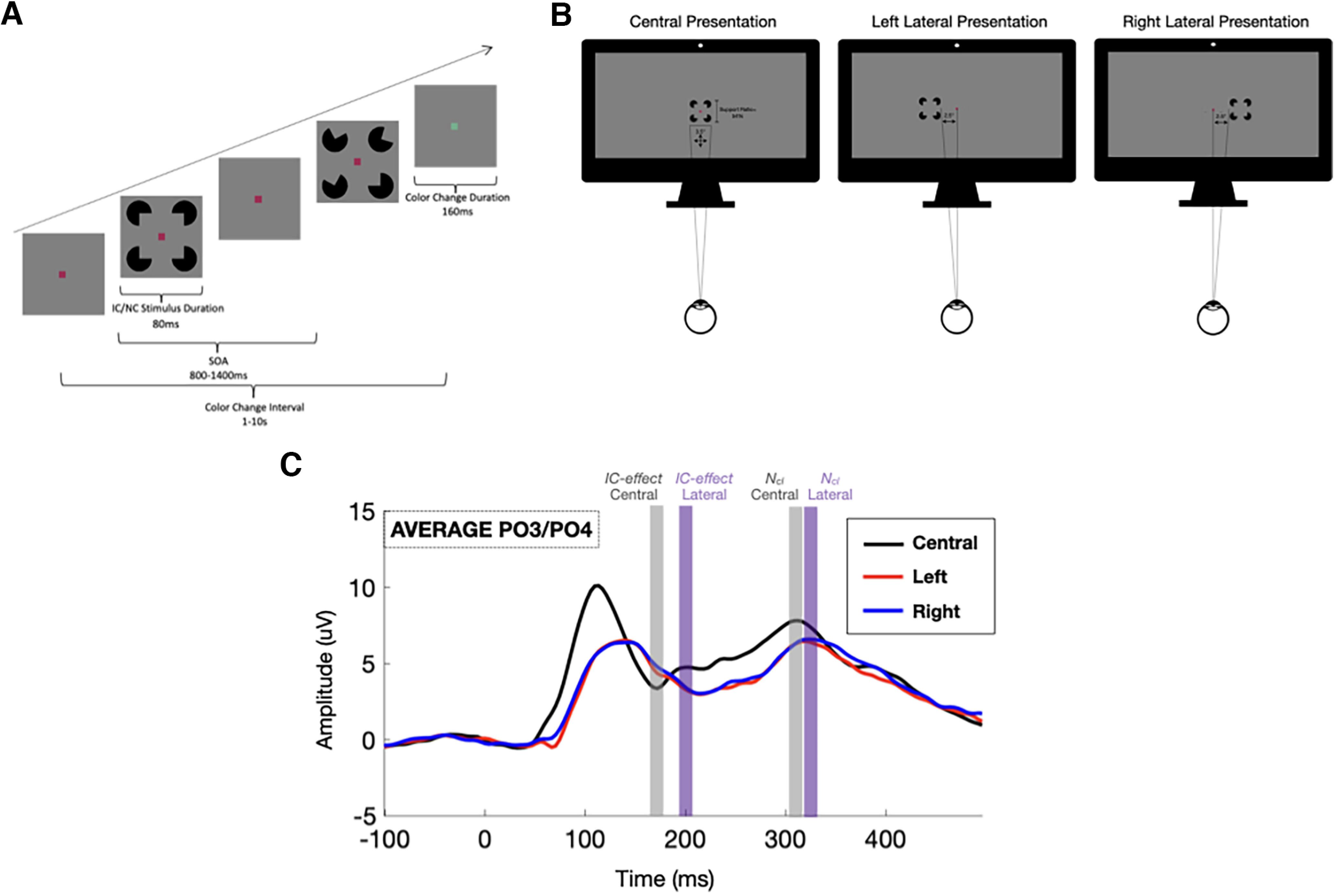
***A***, Schematic representation of experimental paradigm and timing. IC/NC stimuli appear on the screen for 80 ms duration with 800-1400 ms stimulus onset asynchrony (SOA). Central fixation point changes from red to green at variable intervals (1-10 s). ***B***, Schematic representation of central and left and right lateral stimulus presentations. IC/NC stimuli have a support ratio (the ratio of portion of the perimeter occupied by the inducers themselves to the total perimeter of the induced square shape) of 54% and subtend 3.5° of visual angle in both the horizontal and vertical directions. Lateral IC/NC stimuli have an offset of ± 2.5° from the vertical meridian to the nearest edge of the stimulus. ***C***, Components used in the primary analysis as overlaid over the two analyzed bilateral lateral occipital sites (PO3 and PO4), collapsed across all groups (ASD, NT) and stimulus configuration (IC, NC) for the central stimulus presentations.

Stimuli were delivered using Presentation software (version 18.0, Neurobehavioral Systems, www.neurobs.com). A schematic of the experimental paradigm is presented in Figure 1*A*, *B*, and we have made the paradigm code freely available for download with appropriate attribution (Knight et al., 2022b). Individuals were presented with a Kanizsa figure consisting of four Pac-Man-like inducers. Each inducer occupied one of four corners equidistant from a central fixation point. The presence of an IC was defined as alignment of the cut-out of the four inducers such that it collectively produced the image of a square. Conversely, the NC configuration existed when at least one of the cut-outs of the inducers was rotated away from the fixation point and did not produce the illusion of a completed square. The support ratio (the ratio of portion of the perimeter occupied by the inducers themselves to the total perimeter of the induced square shape) was held constant at 0.54. That is, 54% of the observed shape was completed by a real line. When present, illusory shapes subtended 3.5° of visual angle in both the horizontal and vertical planes. The entire stimulus, including inducers, subtended 5.5° in both horizontal and vertical planes. The paradigm included randomly intermixed central and lateral presentations of these stimuli. Laterally presented stimuli were presented at ±2.5° from the vertical meridian to the nearest edge of the stimulus (Fig. 1*B*).

IC or NC stimuli were presented on the screen for a duration of 80 ms with 800-1400 ms randomly jittered onset asynchrony. Participants were instructed to focus on a central red fixation dot (5 × 5 pixels) against a gray background and to press a button on a game controller (SteelSeries 3GC USB 2.0) when the color of the fixation dot changed to green (which occurred on average once every 10 s and lasted ∼160 ms on a random time course uncorrelated with IC/NC presentation) (Fig. 1*A*). The colors were chosen to be equiluminant, with the intention that the change in chromaticity was difficult to perceive without directly foveating. Participants were informed that additional objects may be presented on the monitor but instructed to “do your best to focus on the fixation dot.” There was no specific mention of the nature of the IC and NC stimuli included in the participant instructions. Explicit attention to ICs is not required to elicit electrophysiological indices of IC processing in either adults (Murray et al., 2002) or children (Altschuler et al., 2014), and this paradigm is specifically designed to capture automatic contour integration. An eye-tracker (EyeLink 1000; SR Research) was used to ensure that subjects were fixated on the center of the monitor. A 9-point calibration was performed before each test block, and stimulus onset was paused when the participant's eyes deviated >5° from fixation. The experiment was comprised of seven blocks, each containing ∼180 stimuli with IC/NC configurations and central/lateral locations intermixed and delivered in random order. Participants were allowed breaks as needed between blocks.

### EEG acquisition and preprocessing

All participants sat in a sound-attenuated and electrically shielded booth (Industrial Acoustics) at a distance of 810 mm away from a computer monitor (Acer Predator Z35P 35 inch 21:9 100 Hz) with 1280 × 1024 pixel resolution. EEG data were continuously recorded using a 64-channel Biosemi ActiveTwo acquisition system. The setup includes an analog-to-digital converter and fiber-optic pass-through to a dedicated acquisition computer (digitized at 512 Hz; DC- to-150 Hz pass-band). EEG data were referenced to an active common mode sense electrode and a passive driven right leg electrode. EEG data were processed and analyzed offline using custom scripts that included functions from the EEGLAB (Delorme and Makeig, 2004) and ERPLAB Toolboxes (Lopez-Calderon and Luck, 2014) for MATLAB (The MathWorks). Raw data were downsampled to 256 Hz and filtered between 0.1 and 50 Hz. Bad channels were manually and automatically detected and interpolated using EEGLAB spherical interpolation. The number of interpolated channels and rejected trials are summarized in Table 2. Data were rereferenced to a frontal electrode (Fpz in the 10–20 system convention) and then divided into epochs starting 100 ms before the presentation of each IC/NC stimulus and extending to 500 ms after stimulus onset. Trials containing severe movement artifacts or particularly noisy events were rejected if voltages exceeded ±125 μV. Trials were then averaged to obtain grand average waveforms for central, left, and right IC and NC stimulus presentations for each subject. The *a priori* selection of a frontocentral electrode as a reference was chosen based on extensive prior work demonstrating that the generators for these visual processes are localized to the lateral occipital cortex (Murray et al., 2002, 2004). Use of the frontocentral reference maximizes measurement of VEPs over early visual regions to detect differences across conditions. It was raised during review that including average reference data may aid in comparison of study findings between research groups with different recording setups. For completeness, a *post hoc* comparison of the results between the two types of references was conducted as well. However, we would note that the use of the average reference is not without detractors (Desmedt et al., 1990). While the average reference is valuable in some experimental contexts, the underlying presumption of zero-centered potentials can induce distortion depending on the generator source, particularly under circumstances where there is incomplete electrode coverage of the scalp or, importantly for our child study populations, variable skull shape and thickness.

**Table 2.** Interpolated channels and accepted trials

			NT (*n* = 31)	ASD (*n* = 29)	Significance (*p*)
Interpolated channels, mean (SD)			5.55 (3.4)	3.72 (2.7)	0.027
% accepted trials, mean (SD)	NC	Central	81.8 (13.6)	79.6 (18.6)	0.600
		Left	80.9 (13.7)	80.4 (17.9)	0.908
		Right	80.8 (14.5)	79.4 (18.4)	0.750
	IC	Central	82.6 (13.0)	80.8 (18.1)	0.651
		Left	81.5 (13.8)	78.8 (18.2)	0.526
		Right	81.2 (14.4)	79.6 (17.9)	0.692

SD: standard deviation.

### Statistical analyses

#### Primary analyses

Statistical analyses were implemented in SPSS (IBM SPSS Statistics for MacOS, version 27.0). In order to examine contour integration for centrally and laterally presented Kanizsa figures in ASD and NT participants, while limiting Type II errors, the initial analysis was restricted both spatially and temporally. A pair of bilateral ROIs comprising electrode sites over the lateral occipital cortex bilaterally (PO3 and PO4) were defined based on previous studies showing maximal IC effects over these regions (Senkowski et al., 2005; Kemner et al., 2009). Time windows for the early IC effect and later *N_cl_* VEP components were first broadly defined based on component latency windows described in a previous study mapping the spatiotemporal dynamics of IC processing in NT children (Altschuler et al., 2014) and then further refined within these general component time windows by the grand-averaged waveforms collapsed across both groups, inclusive of IC and NC stimuli (i.e., without regard for or bias from the dependent measure of interest). Mean amplitudes were then computed over a 10 ms time window for the IC effect and *N_cl_* components. Given the expected longer latency of response in IC processing for lateral presentations, time windows were defined separately for central and lateral presentations. For centrally presented stimuli, these encompassed two time windows centered at peak activity (IC effect = 167-177 ms; *N_cl_* = 305-315 ms). For laterally presented stimuli, IC effect and *N_cl_* time windows were delayed to 208-218 and 319-329 ms, respectively (Fig. 1*C*). For each of these components, we first implemented separate mixed-model ANOVAs with a between-subjects factor of group (NT, ASD) and within-subject factors of stimulus configuration (IC, NC), stimulus location (central, left, right), and hemisphere (left PO3, right PO4), with age as a continuous covariate.

#### *Post hoc* analyses

Given the skewed sex ratio in the ASD group, we also explored any effect of sex by conducting a *post hoc* analysis separately on the NT group only. Data from NT participants were analyzed using a mixed-model ANOVA with a between-subjects factor of sex and within-subject factors of stimulus type (IC, NC), stimulus location (central, left, right), and hemisphere (left PO3, right PO4), as well as age as a continuous covariate across the same two time windows used in the primary analysis. Finally, to rule out any group-related differences in timing of the VEP, we tested as a secondary analysis for any difference in N1 latency evoked by centrally presented stimuli across groups. The N1 latency was defined for each individual subject as the time point at which the N1 reached peak negativity for each stimulus type (IC, NC) and hemisphere (left PO3, right PO4), again with age as a continuous covariate.

In order to explore the rich information provided by our high-density electrophysiological dataset more liberally across time and location, we also implemented an exploratory mass univariate analysis across all 64 scalp electrodes and time points between 100 and 300 ms in a 2 × 2 design with a between-subjects factor of group (NT, ASD) and within-subject factors of stimulus configuration (IC vs NC) for centrally presented stimuli. We used the Benjamini and Yekutieli (2001) procedure to control false discovery rate (FDR) at 0.05 (Benjamini and Yekutieli, 2001). This analysis was implemented in the Factorial Mass Univariate Toolbox (Fields, 2017), which extends the existing Mass Univariate Toolbox (Groppe et al., 2011). The approach to calculation of effects in multifactorial designs is based on prior simulation work (Anderson and Braak, 2003), and detailed documentation is provided by the creators of the factorial mass univariate toolbox (https://github.com/ericcfields/FMUT/wiki/Mass-univariate-statistics-and-corrections).

To verify that participants in both groups were equivalently engaged in the color change task during the presentation of the contour stimuli, a supplemental analysis verified that there was no significant difference in accuracy in color change detection as measured by *d*′ (Table 3). In signal detection theory, *d*′ serves as an index of the actual signal relative to the noise and can be measured as the difference between the normalized hit rates and false alarm rates (Green and Swets, 1966).

**Table 3.** Performance on color change detection

Group	*n*	Mean (SD)	Significance
NT	31	4.11 (1.03)	*t*_(58)_ = 0.057, *p* = 0.955
ASD	29	4.09 (0.91)	

SD: standard deviation.

#### Electrophysiologic-phenotypic correlation analysis

To assess the relationship between contour integration processing and perceptual reasoning ability, we conducted two multiple regression analyses separately within each diagnostic group, correlating Full-Scale IQ, Block Design, and Matrix Reasoning domain scores with the IC effect. Next, to assay for attentional impacts on contour integration processing, we conducted a set of linear regression analyses within each diagnostic group comparing SNAP-IV scores with the IC effect. Finally, to assay for relationships between contour integration and autism symptomatology, we conducted a separate multiple regression analysis within each diagnostic group and the Social Responsiveness Scale Total T Score. The IC effect amplitudes used for these analyses were calculated from the IC-NC difference waveforms evoked by centrally presented stimuli in the N1 time window (averaged across the PO3 and PO4 electrode sites).

## Results

**Figure 2.**
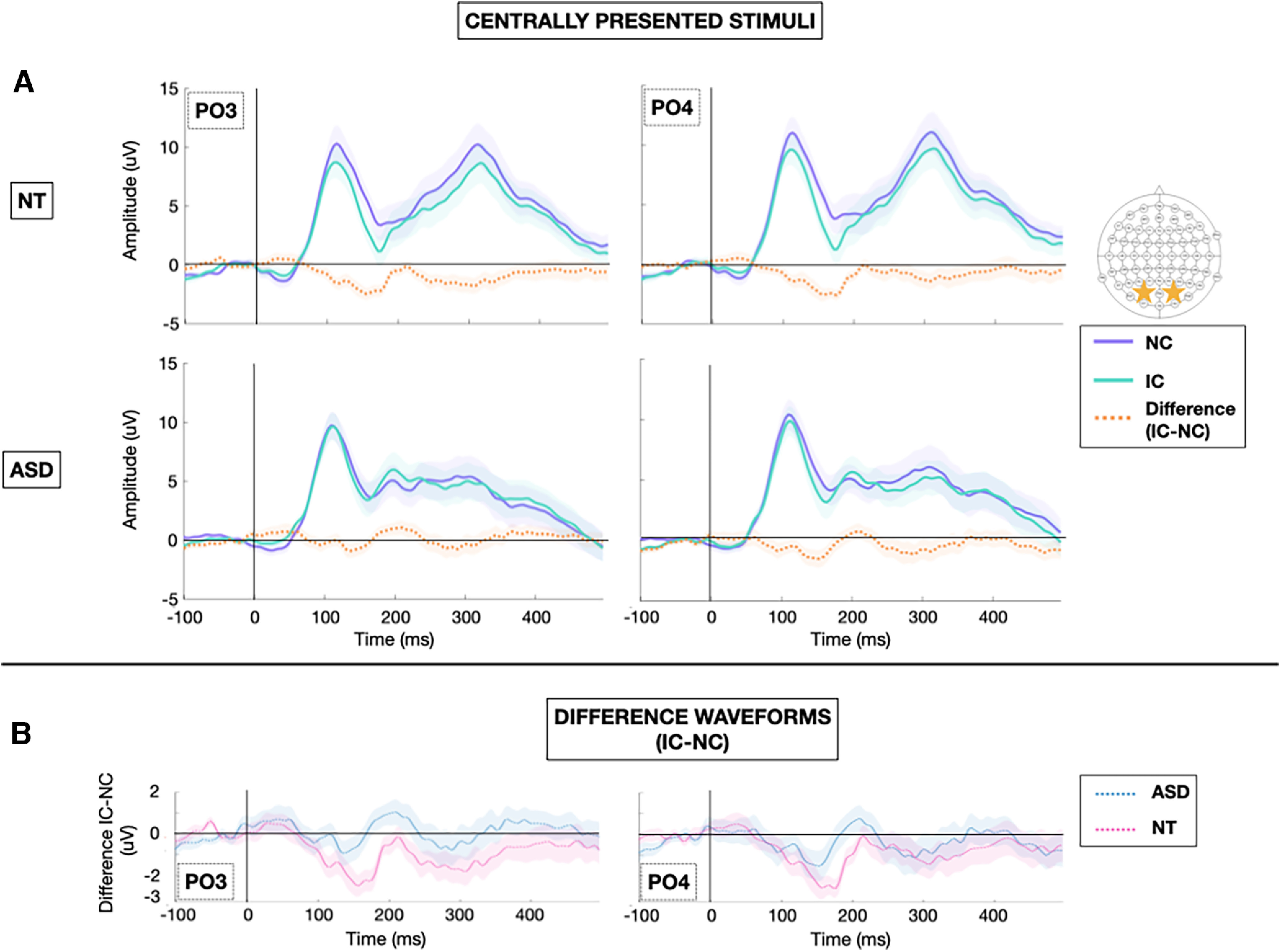
***A***, Grand average VEPs for centrally presented stimuli at bilateral lateral occipital sites (PO3 and PO4) obtained in NT (top) and ASD (bottom) participants to NC (purple), and IC (green), and IC-NC difference potential (orange). Shaded regions represent ± standard error of the mean (SEM). ***B***, Difference waveforms (IC-NC) obtained in ASD (blue) and NT (pink) participants at bilateral lateral occipital sites (PO3 and PO4).

**Figure 3.**
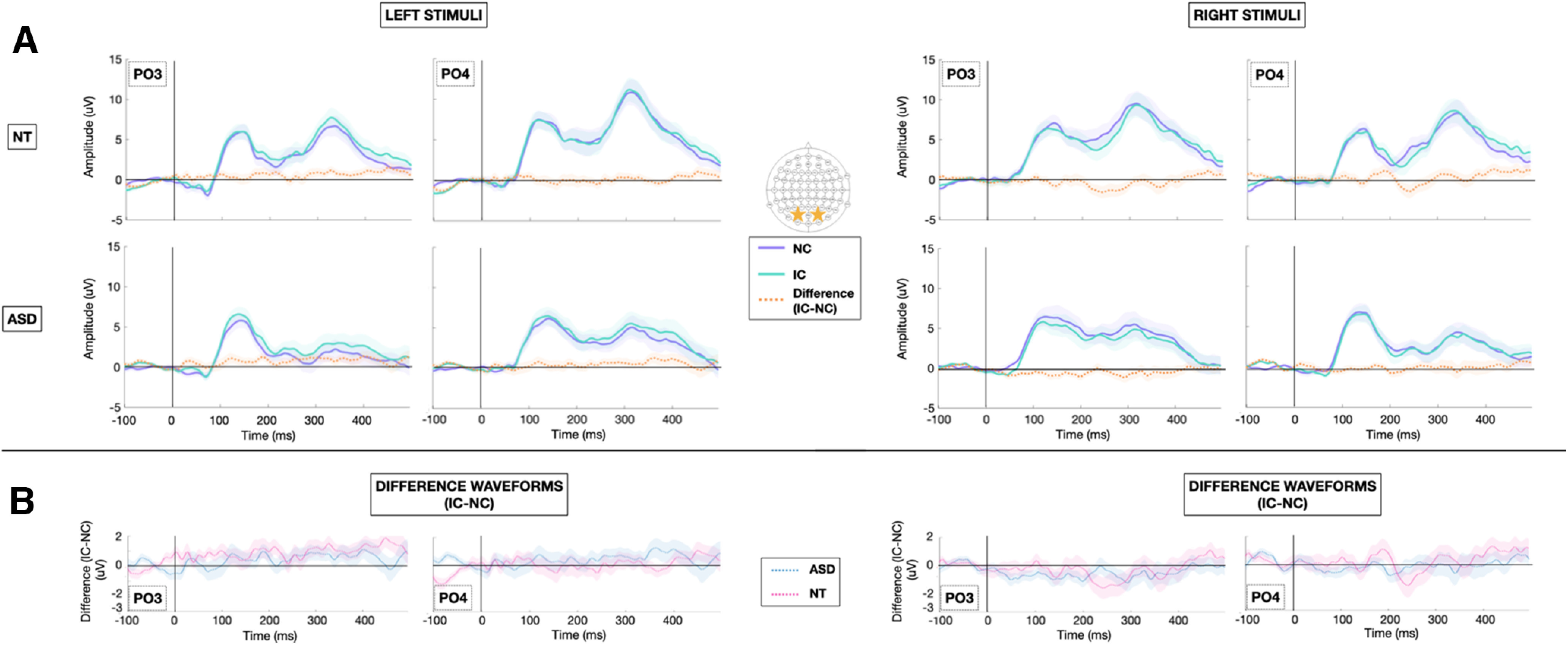
***A***, Grand average VEPs for laterally presented stimuli at bilateral lateral occipital sites (PO3 and PO4) obtained in NT (top) and ASD (bottom) participants to NC (purple), and IC (green), and IC-NC difference potential (orange). Shaded regions represent ± standard error of the mean (SEM). ***B***, Difference waveforms (IC-NC) obtained in ASD (blue) and NT (pink) participants at bilateral lateral occipital sites (PO3 and PO4).

**Figure 4.**
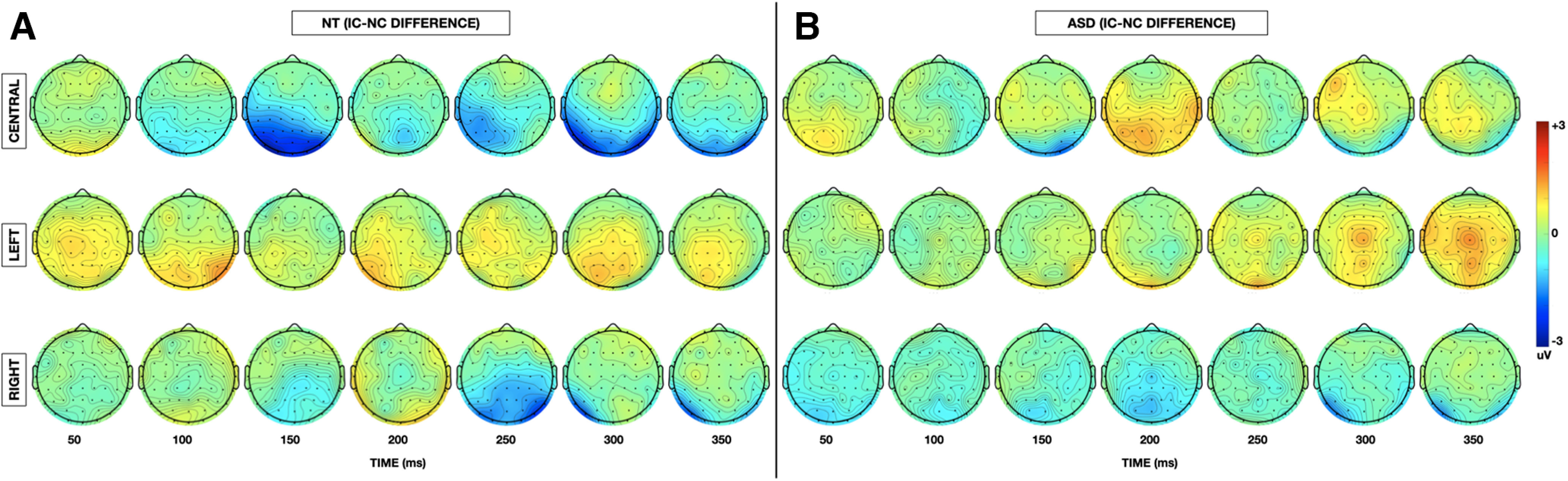
***A***, Topographic representation of the difference in instantaneous amplitude of evoked response between IC and NC stimuli for central (top), left (middle), and right (bottom) stimulus locations for NT participants at 50 ms intervals between 100 and 350 ms after stimulus onset. ***B***, Topographic representation of the difference in instantaneous amplitude of evoked response between IC and NC stimuli for central (top), left (middle), and right (bottom) stimulus locations for ASD participants at 50 ms intervals between 50 and 50 ms after stimulus onset.

Grand-average VEPs to the IC and NC stimulus configurations at the *a priori* defined electrodes of interest (PO3 and PO4) are depicted for each group in Figure 2 (centrally presented stimuli) and Figure 3 (laterally presented stimuli). To highlight contour integration, the difference in evoked response to IC minus NC stimuli is also represented for each group at each electrode of interest. Topographic maps depicting the differences in evoked response to IC minus NC stimuli for central and lateral presentations in each of the NT and ASD groups allow for visualization of effects across the entire array (Fig. 4).

### IC effect

**Figure 5.**
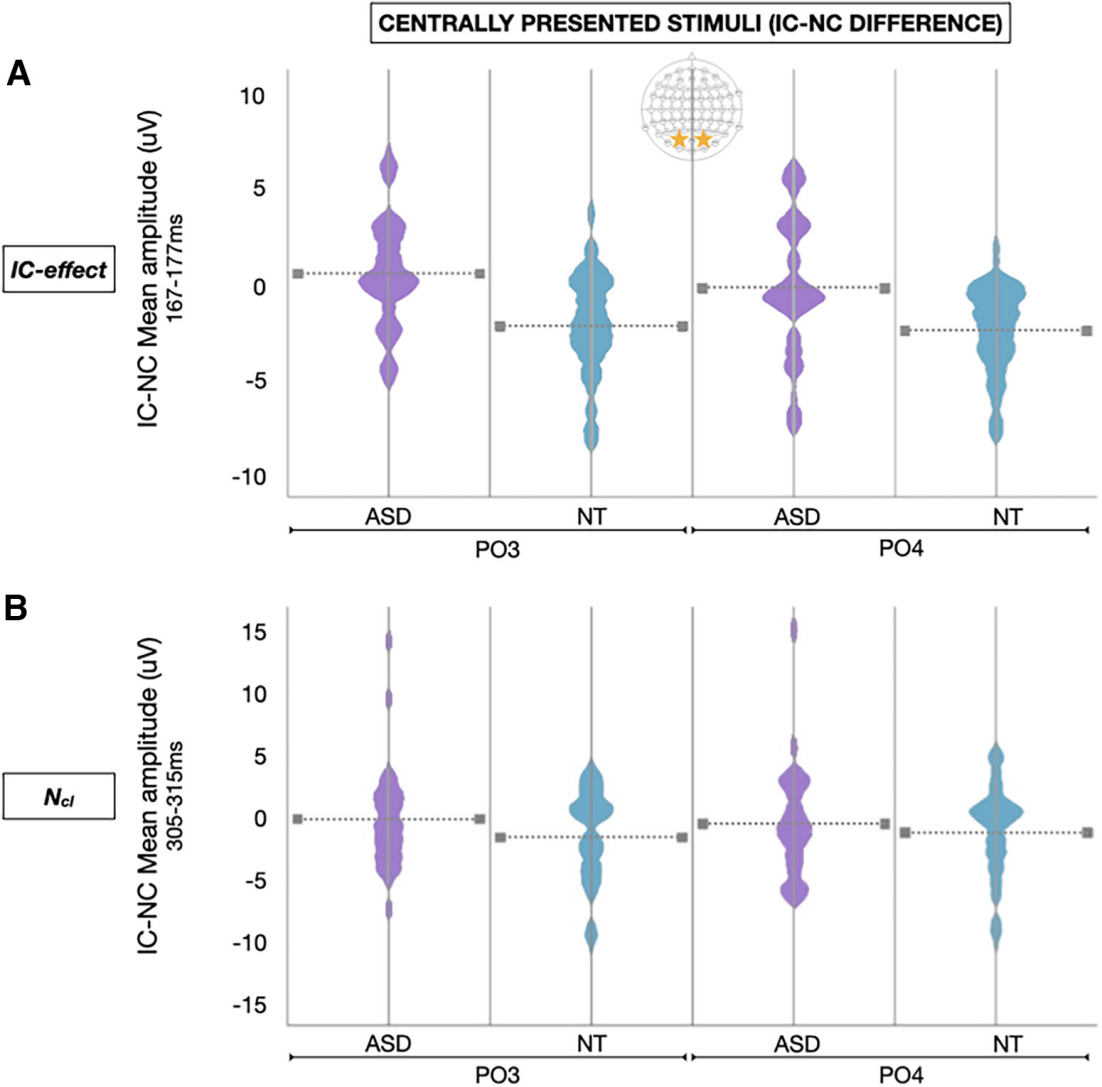
***A***, Violin plots represent the distribution of the difference in IC-NC mean amplitude (μV) for centrally presented stimuli during the IC effect time window (167-177 ms) at bilateral lateral occipital sites (PO3 and PO4) among ASD (purple) and NT (blue) participants. ***B***, Violin plots represent the distribution of the difference in IC-NC mean amplitude (μV) for centrally presented stimuli during the *N_cl_* time window (305-315 ms) at bilateral lateral occipital sites (PO3 and PO4) among ASD (purple) and NT (blue) participants. Horizontal dotted lines represent the group mean.

The results of the primary analysis are summarized in Table 4. As evident in Figure 5, for the IC effect time windows, there was a group × stimulus configuration × stimulus location interaction (*F*_(2114)_ = 5.746, *p* = 0.004, η_p_^2^ = 0.092). For the central stimulus presentation, NT individuals demonstrated a strong IC effect characterized by greater negativity in response to the IC versus NC stimulus configuration. The IC effect was absent in NT participants for laterally presented stimulus configurations. In contrast, ASD individuals demonstrated no IC effect regardless of stimulus location. As expected, there was also a stimulus location × hemisphere interaction (*F*_(1.177,67.063)_ = 4.865, *p* = 0.025, η_p_^2^ = 0.079, Greenhouse-Geisser–corrected) whereby both hemispheres showed roughly equivalent activation in response to central stimulus presentations, whereas lateral stimulus presentations evoked greater magnitude visual potentials in the contralateral hemisphere. There were no main effects of group, stimulus configuration, stimulus location, or hemisphere; and no other interactions were significant after controlling for age.

**Table 4.** Primary analysis results summary

Main effects	IC effect (*p*)	*N_cl_* (*p*)
Group	0.695	0.028*
Hemisphere	0.595	0.269
Stimulus configuration (IC/NC)	0.278	0.288
Stimulus location (central/left/right)	0.833*^a^*	0.555
Within-subject interactions		
Hemisphere × stimulus configuration	0.699	0.732
Hemisphere × stimulus location	0.025^a^*	0.001^a^**
Stimulus configuration × stimulus location	0.619	0.506
Hemisphere × stimulus configuration × stimulus location	0.558	0.062*^a^*
Within-subject × between-subject interactions		
Hemisphere × group	0.588	0.481
Stimulus configuration × group	0.449	0.811
Stimulus location × group	0.234*^a^*	0.743
Hemisphere × stimulus configuration × group	0.269	0.314
Hemisphere × stimulus location × group	0.798*^a^*	0.996*^a^*
Stimulus configuration × stimulus location × group	0.004**	0.269
Hemisphere × stimulus configuration × stimulus location × group	0.106	0.747*^a^*
Covariates		
Age	0.551	0.498

*^a^*Greenhouse-Geisser–corrected for violation of Mauchly's test of sphericity.

*p < 0.05;

**p < 0.01; *n*_NT_ = 31, *n*_ASD_ = 29.

### N_cl_

For the *N_cl_* component, there was a main effect of group (*F*_(1,57)_ = 5.077, *p* = 0.028, η_p_^2^ = 0.082) with NTs showing greater magnitude VEPs than ASD participants during the *N_cl_* time windows. There was also a continuation of the stimulus location × hemisphere interaction observed during IC effect time windows (*F*_(1.430,81.531)_ = 8.711, *p* = 0.001, η_p_^2^ = 0.133, Greenhouse-Geisser–corrected) whereby lateral stimulus presentations evoked greater magnitude visual potentials in the contralateral hemisphere. However, there were no group differences related to stimulus configuration (IC vs NC); neither group demonstrated significant *N_cl_* response to the IC versus NC stimuli after controlling for age (Fig. 5). No other main effects or interactions were significant.

### *Post hoc* analyses

To assess the likelihood that ASD-related findings observed in the IC effect time window would have been driven by differences in sex distribution between the ASD and NT groups, we assessed for any sex-related influences on contour integration in the IC effect time window via a *post hoc* mixed-design ANOVA comparing males and females within the NT group only (Table 5). Within the NT group, there was no effect of sex nor any sex-related interactions significantly influencing the strength of the IC effect. As a final *post hoc* analysis, we assessed for any group-related differences in latency of the N1 evoked by centrally presented stimuli across groups (Table 6). There was no difference in N1 latency between ASD and NT groups for either hemisphere or stimulus type (NC or IC). Younger age was associated with longer N1 latency (*F*_(1,57)_ = 12.574, *p* < 0.001, η_p_^2^ = 0.181).

**Table 5.** Secondary analysis results summary: sex-related effects in NTs*^a^*

	IC effect (*p*)
Main effects	
Sex	0.608
Within-subject × between-subject interactions	
Hemisphere × sex	0.529
Stimulus configuration × sex	0.092
Stimulus location × sex	0.115
Hemisphere × stimulus configuration × sex	0.741
Hemisphere × stimulus location × sex	0.100
Stimulus configuration × stimulus location × sex	0.355
Hemisphere × stimulus configuration × stimulus location × sex	0.861

*^a^n*_female_ = 21, *n*_male_ = 10.

**Table 6.** Secondary analysis results summary: N1 latency

	N1 latency (*p*)
Main effects	
Group	0.585
Hemisphere	0.474
Stimulus configuration (IC/NC)	0.176
Within-subject interactions	
Hemisphere × stimulus configuration	0.087
Within-subject × between-subject interactions	
Hemisphere × group	0.112
Stimulus configuration × group	0.245
Hemisphere × stimulus configuration × group	0.084
Covariates	
Age	<0.001**

**p < 0.01; *n*_NT_ = 31, *n*_ASD_ = 29.

**Figure 6.**
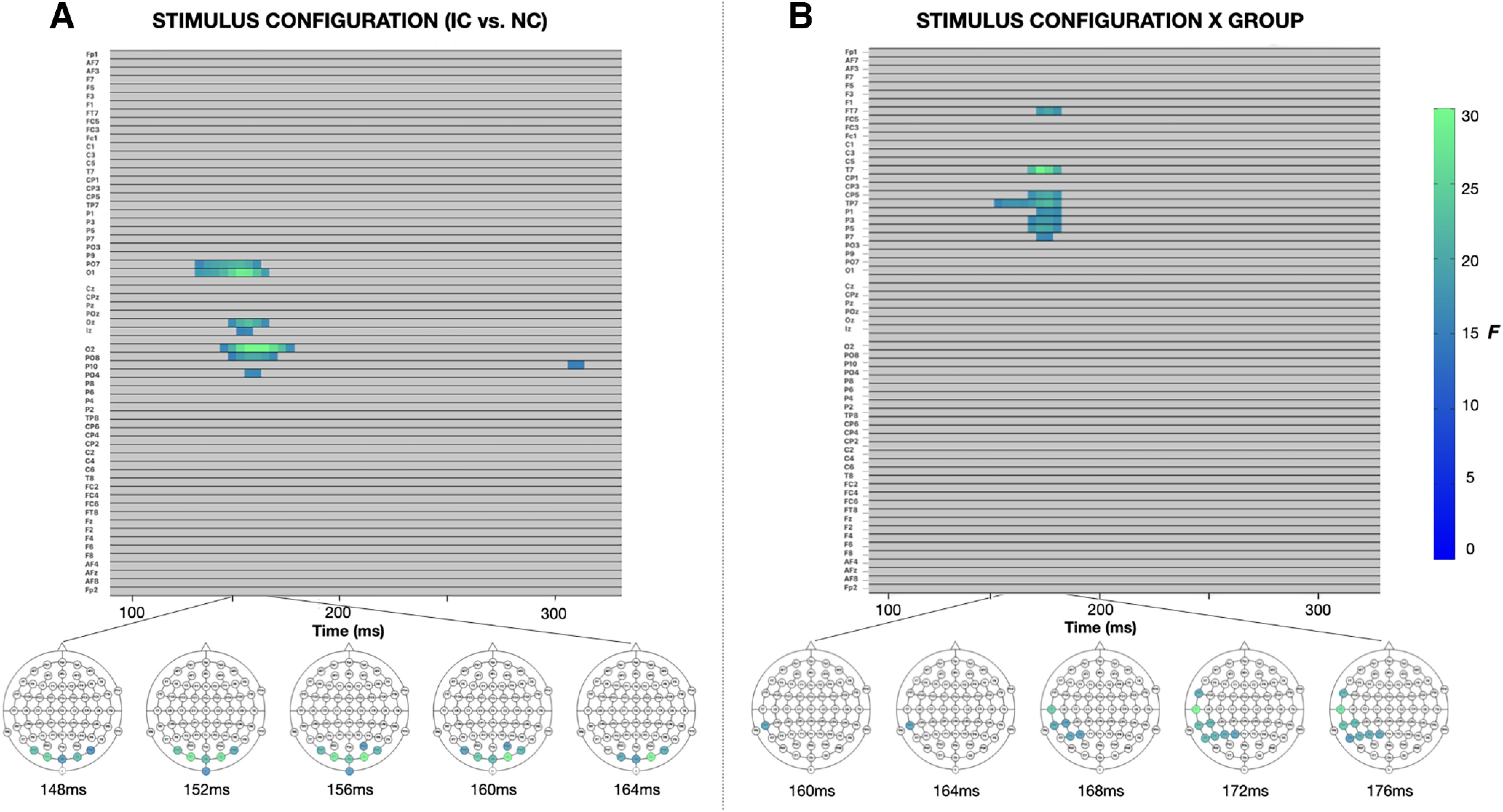
Results of the exploratory factorial mass univariate analysis across all electrodes and time points between 100 and 300 ms with a between-subjects factor of group (NT, ASD) and within-subject factor of stimulus configuration (IC, NC). ***A***, Regions and time points demonstrating a main effect of stimulus configuration. ***B***, Regions and time points demonstrating a group × stimulus configuration interaction. There was no significant main effect of group.

As the initial *a priori* defined analysis was restricted in both space and time, we conducted an exploratory analysis to examine for group differences in contour integration of centrally presented stimuli across the whole brain over a more liberal timespan. Topographic maps depicting the differences in evoked response to IC minus NC stimuli for central and lateral presentations in each of the NT and ASD groups allow for visualization of effects across the entire array (Fig. 4). This secondary analysis replicated was consistent again with prior literature in demonstrating that IC processing is most pronounced between 150 and 180 ms over the midline and bilateral occipital regions (significant electrodes at *t* = 156 ms: PO7, O1, Oz, Iz, O2, PO8, PO4) (Fig. 6*A*). In addition, this analysis highlighted a group × stimulus configuration in the left frontal-temporal and parietal regions (significant electrodes at *t* = 172 ms: FT7, T7, CP5, TP7, P1, P3, P5, P7) during this same IC effect time window whereby individuals with autism demonstrated an attenuated negative response to IC versus NC stimuli in this region in addition to the lateral occipital region revealed in the primary analysis (Fig. 6*B*). There was no main effect of group at any of the electrodes or windows tested.

**Figure 7.**
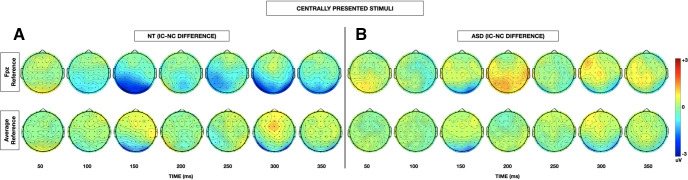
Topographic representation of the difference in instantaneous amplitude of evoked response between IC and NC stimuli for central stimulus locations comparing the FPz frontal reference electrode (top) with an average reference (bottom) for NT (***A***) and ASD (***B***) participants at 50 ms intervals between 100 and 350 ms after stimulus onset.

Finally, in response to a reviewer suggestion that presentation of data with respect to an average reference may be of additional value to aid in comparison of study findings between research groups with different recording setups, topographic maps of the difference in IC-NC amplitude evoked by centrally presented stimuli using an average reference are depicted in Figure 7. The primary analysis results are compared between the two types of references in Table 7. For a brief discussion of the relative merits of the different reference types, see EEG acquisition and preprocessing.

**Table 7.** Comparing average reference to frontal reference

	IC effect (*p*) (average reference)	IC effect (*p*) (Fpz reference)
Group	0.256	0.695
Hemisphere	0.025*	0.595
Stimulus configuration (IC/NC)	0.263	0.278
Stimulus location (central/left/right)	0.632*^a^*	0.833*^a^*
Within-subject interactions		
Hemisphere × stimulus configuration	0.330	0.699
Hemisphere × stimulus location	0.121*^a^*	0.025^a^*
Stimulus configuration × stimulus location	0.814*^a^*	0.619
Hemisphere × stimulus configuration × stimulus location	0.621*^a^*	0.558
Within-subject × between-subject interactions		
Hemisphere × group	0.352	0.588
Stimulus configuration × group	0.636	0.449
Stimulus location × group	0.494*^a^*	0.234*^a^*
Hemisphere × stimulus configuration × group	0.742	0.269
Hemisphere × stimulus location × group	0.503*^a^*	0.798*^a^*
Stimulus configuration × stimulus location × group	0.074	0.004**
Hemisphere × stimulus configuration × stimulus location × group	0.247	0.106
Covariates		
Age	0.299	0.551

*^a^*Greenhouse-Geisser–corrected for violation of Mauchly's test of sphericity.

*p < 0.05;

**p < 0.01; *n*_NT_ = 31, *n*_ASD_ = 29.

### Electrophysiologic-phenotypic correlations

**Figure 8.**
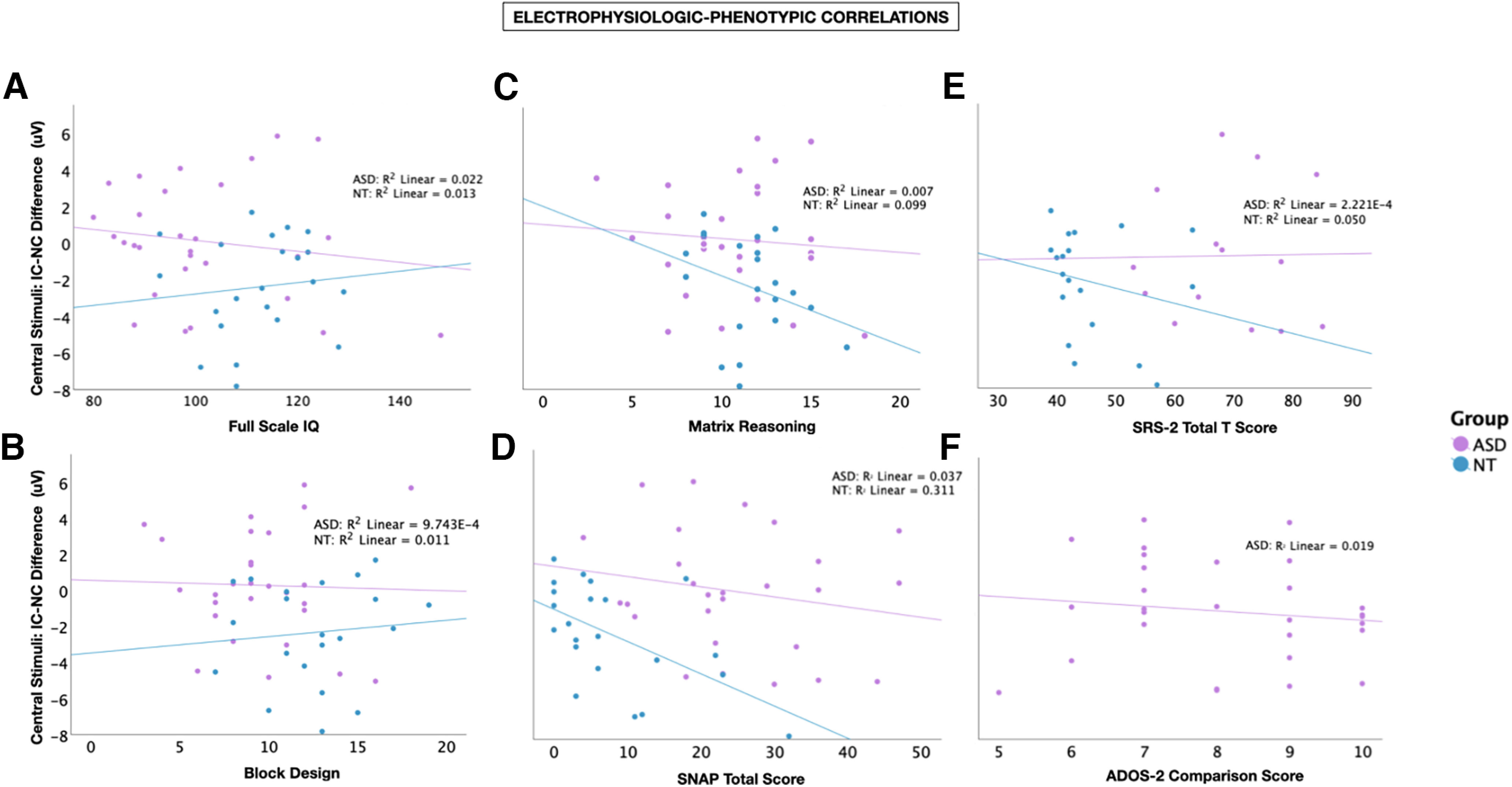
Scatter plot represents in ASD (purple) and NT (blue) participants the relationship between difference in IC-NC mean amplitude (μV) for centrally presented stimuli during the IC effect time window and (***A***) Full-Scale IQ, (***B***) Block Design scaled score, (***C***) Matrix Reasoning scaled score, (***D***) SNAP-IV measure of ADHD symptoms, (***E***) SRS-2 Total T Score, and (***F***) ADOS-2 Comparison Score (ASD participants only).

There were no relationships between the magnitude of the IC effect and Full-Scale IQ, Block Design, or Matrix Reasoning subscales in either diagnostic group (NT: *F*_(3,21)_ = 1.415, *p* = 0.271, adjusted *R*^2^ = 0.056; ASD: *F*_(3,27)_ = 0.066, *p* = 0.978, adjusted *R*^2^ = –0.116) (Fig. 8*A–C*). A stronger IC effect was associated with elevated parent-reported symptoms of inattention, hyperactivity, and impulsivity (as measured by the SNAP-IV) in the NT group only (NT: *F*_(1,21)_ = 9.032, *p* = 0.007, *R*^2^ = 0.311; ASD: *F*_(1,26)_ = 0.973, *p* = 0.333, *R*^2^ = –0.037) (Fig. 8*D*). There was no relationship between the magnitude of the IC effect and SRS-2 Total T score in either group (NT: *F*_(3,21)_ = 1.415, *p* = 0.271, adjusted *R*^2^ = 0.056; ASD: *F*_(3,27)_ = 0.066, *p* = 0.978, adjusted *R*^2^ = –0.116) (Fig. 8*E*). The ADOS-2 was completed only for participants in the ASD group and is not included in the analysis, but a scatter plot depicting the relationship between the magnitude of the IC effect and ADOS-2 comparison scores is included in Figure 8*F* in response to a reviewer suggestion.

## Discussion

### Centrally presented IC processing

Children with ASD demonstrated a marked attenuation of the early and automatic contour integration that is present in NT children viewing centrally presented IC stimuli. This finding provides clear electrophysiologic evidence of disordered contour integration in children with ASD, extending beyond the preschool age cohort previously studied by Stroganova et al. (2007). Thus, despite several behavioral studies suggesting that children with ASD can accurately perceive ICs (Milne and Scope, 2008; Hadad et al., 2019; Gowen et al., 2020), accumulating evidence points to clear differences in underlying neurophysiologic mechanisms that appear early in development and persist across childhood and adolescence.

Contour integration has been demonstrated in a variety of work to be dependent on feedback connections from higher-order sensory cortices (Murray et al., 2002; Halgren et al., 2003; Altschuler et al., 2012; Shpaner et al., 2013; Wokke et al., 2013). Typically, ambiguous sensory inputs, like those of ICs, are shaped by statistical predictions about configuration acquired through prior exposure, processes that are thought to be conveyed by feedback circuitry (Lee and Mumford, 2003). The ultimate percept in typical visual processing therefore reflects a balance of feedforward and feedback processing. Initial input to V1 is detectable at ∼55 ms after stimulus presentation (Foxe and Simpson, 2002; Kelly et al., 2008), so at this latency a measurement would reflect largely feedforward information and, given the receptive field properties of V1 neurons, low-level featural information. During the N1 latency (beginning ∼100 ms later), assays have convincingly implicated feedback circuitry (Lamme et al., 1998a; Hupe et al., 2001; Angelucci et al., 2002). The initial pass of feedforward information from V1 reaches dorsolateral frontal cortex in ∼30 ms (Foxe and Simpson, 2002). Considering this, it is not hard to see how quickly the influence of feedback may permeate every level of the visual hierarchy (Zipser et al., 1996; Lamme et al., 1998a), and indeed this has been convincingly demonstrated in the integration of IC stimuli (Wokke et al., 2013; Pak et al., 2020; Zeng et al., 2020). Therefore, diminished electrophysiologic evidence of contour integration in ASD implies a reduction in the ability to deploy this higher-order feedback information in support of early visual processing. Prior investigation has demonstrated that VEPs are robust and highly reproducible, arguing against a neural unreliability in autism and indicating that group differences generally cannot be accounted for simply by increased variability in the ASD relative to NT group (Butler et al., 2017). Thus, this study provides convincing empiric evidence of disordered feedback processing in ASD and offers a conceptual link between many prominent ASD theories, including weak central coherence, superior local and reduced global processing, and predictive coding deficits (Bertone et al., 2005; Mottron et al., 2006; Happe and Booth, 2008; Pellicano and Burr, 2012). Each of these theories postulates a diminished ability to apply global contextual features, experience, and/or expectation to facilitate efficient sensory perception in a complex and rapidly changing environment, and thus depends on timely and robust integration of higher-order feedback with incoming sensory information.

Although not explicitly accounted for by other predominant theories of weakened top-down input to lower-level sensory processing regions, an additional higher-order cognitive process that is increasingly important to consider is the possible modulating role of attention on processing in primary sensory cortices in children with developmental disabilities. Selective attention operates at multiple levels in the visual system and appears to be mediated by higher feedback to hierarchically early visual regions (Hopfinger et al., 2004). Although the relationship between attentional abnormalities and ASD phenotypes is incompletely understood, impairments in selective attention and attentional disengagement within multiple sensory modalities have been described in ASD (Ciesielski et al., 1990; Landry and Bryson, 2004; Kawakubo et al., 2007; Keehn et al., 2013; Sacrey et al., 2014). In this study, electrophysiologic measures were recorded during a task in which ICs were passively viewed while attention was explicitly directed to an alternative task of identifying color change. Therefore, one possible interpretation is that attenuated IC processing in the ASD group may reflect, in part, a tendency to overfocus on the color change task with less allocation of attentional resources toward the IC stimuli. A recent investigation involving other visual tasks has indicated a modulating role of attention in contributing to electrophysiological visual processing deficits observed in ASD, whereby children with ASD may have specific deficits in automatic processing that can be partially to completely ameliorated by explicit attention to processing these stimuli (Knight et al., 2022a). Potentially supportive of this possibility are the ASD-associated differences in contour integration that extend beyond the lateral occipital region into the left frontotemporal and parietal regions that were identified here through exploratory analysis, although this finding should be interpreted with much caution given the *post hoc* design. Although outside the scope of this study, additional investigation to evaluate neural processing of ICs under varied attention conditions would therefore be of much interest.

It does not appear from these results that the development of contour integration is merely delayed in ASD children compared with chronologically age-matched peers. Younger participants across both groups in the study tended to demonstrate higher-amplitude VEPs, which is consistent with a wide body of previous research and thought to be related to developmental maturation of electrophysiologic mechanisms and/or age-related changes in skull thickness affecting volume conduction (Adeloye et al., 1975). However, even the youngest NT participants in our study demonstrated evidence of IC processing, echoing findings from previous studies on the developmental trajectory of contour integration that this phenomenon matures relatively early (Brandwein et al., 2011, 2013; Altschuler et al., 2014). Additionally, age did not significantly interact with diagnostic group nor with stimulus configuration suggesting that the magnitude of the group differences in IC effect were consistent across all ages studied. Finally, both groups showed a trend toward some limited contour integration in the *N_cl_* timeframe that did not reach significance after correcting for age and was not substantially different across groups. In younger NT children, more pronounced conceptual stage processing has been described as a marker of developmental maturity that subsequently decreases with age (Altschuler et al., 2014). The ASD children in this study did not demonstrate this immature pattern of an overreliance on later effortful conceptual phase processing as compensation for attenuation in early automatic IC processing. Thus, the similarity in processing during the *N_cl_* period regardless of diagnosis provides further evidence against simple developmental immaturity of contour integration mechanisms in children with ASD. Of note, the majority of the participants were between age 10 and 17 years old with relatively few participants (*n* = 8) in the youngest end of the age range between 7 and 9 years old, which may impact study sensitivity to detect more subtle group differences in developmental trajectory during this early period. However, additional evidence against intact but slower more inefficient IC processing in ASD is provided by the equivalency in latency of VEP components between groups. Irrespective of group, younger age was associated with longer N1 latency, and this was not more exaggerated in the ASD group. Thus, the results are more suggestive of deviant rather than delayed development of early automatic contour processing in children with ASD across a broad age range. Furthermore, we did not observe any evidence the IC effect was intact while the *N_cl_* was selectively attenuated in ASD. Certainly, closure deficits with more complex object completion cannot be ruled out. Indeed, one would predict given the very early disruption in feedback processing underlying the IC effect observed here in combination with prior psychophysical study of object closure in autism (Dehaqani et al., 2016; Arun, 2022), that neural processing in more complex visual object recognition is likely also impacted. Based on the disrupted IC effect and the strong dependence on feedback systems in object closure and figure-ground separation implied by previous studies (Lamme et al., 1998a,b, 2002; Lamme and Roelfsema, 2000; Lamme and Spekreijse, 2000; Layton et al., 2014; Park et al., 2022), a logical next step would be to more closely examine perceptual closure as well as object segmentation. To specifically test the robustness of these processes, more complex stimuli would be needed (Dehaqani et al., 2016; Arun, 2022).

Notably, the IC effect in the school-age and adolescent ASD children in this study was absent as opposed to inverted as has been described previously in preschool age ASD children (Stroganova et al., 2007). This apparent discrepancy in the degree of atypicality between studies may be attributable to age-related developmental changes, but it is possible that alterations in paradigm or EEG preprocessing could contribute to some of the differences across studies as well. For instance, IC perception is thought to be largely scale invariant but does depend on other stimulus characteristics, such as the support ratio and exposure duration (Liinasuo et al., 1997). Despite this, both studies identified clear electrophysiologic evidence of disordered automatic contour integration in ASD participants. These findings may contrast with those obtained via imaging, as an fMRI study using similar Kanizsa figures found no difference between adolescents with ASD and NT controls in patterns of primary visual cortex activation evoked by IC versus NC stimuli (Utzerath et al., 2019). This difference across studies involving different methodologies could possibly be explained either by the superior temporal resolution of EEG and/or by the localization of ASD-associated deficits more toward higher-order visual cortices, such as lateral occipital cortex as opposed to primary visual cortex. Performing both imaging and electrophysiology in the same subjects would therefore be illuminating to help afford a more comprehensive picture of contour integration mechanisms in ASD.

### Laterally presented IC stimuli

When stimuli were presented lateral to the fixation point, neither NT nor ASD children demonstrated early automatic IC processing in the N1 timeframe as indexed by the IC effect. However, there was some evidence of a trend toward contour integration emerging later in time (∼250 ms) in NT children only for stimuli presented to the right of fixation. Since laterally presented stimuli are more challenging to process and in adults result in greater evidence of effortful processing, the most parsimonious explanation is that the lack of difference between groups here reflects a floor effect. Without clear consistent evidence of contour integration during this time window in NT children, it would be near impossible to detect any subtle ASD-related deficits. An alternative explanation would be that mechanisms that support processing of laterally presented IC are intact in ASD while mechanisms supporting processing of centrally presented stimuli are selectively disrupted. As NT adults do show evidence of feedback-supported automatic IC processing in the *N_cl_* period for laterally presented stimuli (Murray et al., 2002; Senkowski et al., 2005), comparing NT and ASD adults may be more illuminating to probe these mechanisms.

### Hemispheric lateralization of contour integration

Cortical source localization was not performed in this study, so any estimation of cortical generators represents an extrapolation from data on NT children (Altschuler et al., 2014). However, consistent with the greater body of electrophysiologic research on contour integration, the IC effect was most pronounced at electrodes overlying the lateral occipital cortex among both groups of children in this study. For centrally presented stimuli, there was a trend toward more pronounced IC-specific activation over the left hemisphere in both the NT and ASD groups. There remains some controversy over the pattern of hemispheric lateralization in IC processing. While some studies have suggested a right-sided lateralization for IC processing (Hirsch et al., 1995; Atchley and Atchley, 1998; Larsson et al., 1999; Brighina et al., 2003; Halgren et al., 2003; Seghier and Vuilleumier, 2006), which would be consistent with right hemispheric specialization for perceptual grouping and feature integration (Atchley and Atchley, 1998; Evans et al., 2000; Han et al., 2002), left-sided lateralization of early IC processing similar to that observed here has been previously reported in NT adults (Proverbio and Zani, 2002). Likewise, studies of split-brain patients or those with right hemisphere lesions help clarify these conflicting results through provision of evidence that the right hemisphere does not have a preferential role in contour integration but instead may be more involved in other aspects of perception (i.e., amodal perception) (Corballis et al., 1999; Olk et al., 2001; Vuilleumier et al., 2001). As expected, within both groups, laterally presented stimuli preferentially activated the contralateral hemisphere. Likewise, for laterally presented stimuli, contour integration was more pronounced when stimuli were presented on the right, again suggestive of possible left-sided dominance in contour integration among participants in this study. Importantly for the questions of interest in this study, there were no hemisphere × group interactions, indicating that there was no significant difference in lateralization between groups. Thus, while several studies have implicated atypical lateralization among individuals with ASD in language, motor, or visual perception (Jeste and Nelson, 2009; Fiebelkorn et al., 2013; Kroger et al., 2014; Luckhardt et al., 2014; Keehn et al., 2015; Floris et al., 2016, 2021), this does not appear based on our findings to play a role in IC perception.

### Electrophysiologic-phenotypic correlations

Given that contour integration is a process that involves visuospatial perception, we tested for any relationship the strength of the IC effect and performance on block design and matrix reasoning subtests as well as Full-Scale IQ. The magnitude of the IC effect did not correlate with any of these measures. Thus, the reduction in IC effect among autistic children does not seem to be related to cognitive delays. Of note, IQ testing is a relatively broad measure of cognition; and it remains possible that more detailed neuropsychological investigation could reveal more domain-specific brain–behavior relationships. Additionally, all participants in this study had a Full-Scale IQ > 70, which was deemed a necessary inclusion criterion given the task demands. However, this does limit the range over which cognitive phenotypes may have varied, making it more difficult to detect any relationship between IC processing and cognitive factors. Likewise, we did not observe relationships between contour integration and social communication ability as measured by the SRS-2, although again it is important to note that all participants in the study were fluent verbal communicators, which limits generalizability to more severe subtypes of ASD. There was an apparent relationship between augmented contour integration and elevated symptoms of inattention, hyperactivity, and impulsivity in NT participants only (though no NT participants had received a medical diagnosis of ADHD). No such effect was noted in the ASD group, making it unlikely that comorbid ADHD symptoms in the patient group contributed to the observed findings. However, as discussed above, it would be interesting to assay contour integration processing under varied attention conditions in children with ASD and in children with other neurodevelopmental disorders (e.g., ADHD, learning disability, intellectual disability).

### Endophenotypic implications

There has been much interest in defining objective endophenotypes that may link the behavioral phenotype we recognize as ASD with discrete alterations in neurophysiology. Thus, the electrophysiologic demonstration of an attenuated IC effect as well as similar electrophysiologic deficits observed in other types of sensory perception (Jeste and Nelson, 2009; Luckhardt et al., 2014) indicates the usefulness of electrophysiology in detecting subtle visual perceptual changes even when not apparent in behavioral measures. As many of these types of visual processing mechanisms, including contour integration, mature over early childhood (Altschuler et al., 2014), monitoring trajectories across this critical intervention period may afford a useful window into perceptual and cognitive development.

### Limitations

The paradigm was designed to elicit automatic processing of ICs without explicit attention directed to contour presence or absence. We did attempt to control for some aspects of overt attention through the use of infra-red eye-tracking to ensure gaze did not deviate from the central fixation dot. However, given the primacy of IC perception, it is certainly possible that some participants noticed the presence of ICs, resulting in covert attentional reorienting. This makes it difficult to completely ensure that the data reflect only unattended contour integration. Additionally, the participants in the study represent a relatively narrow range of the phenotypic variation that characterizes ASD. As noted above, given the requirement for children to understand task directions and provide behavioral responses, only children with average to above average intelligence and fluent verbal ability participated. This limits the generalizability of the findings to children with comorbid intellectual disability and/or severe language impairment. Although outside the scope of this study, future adaptations of the paradigm to eliminate requirements for a specific behavioral response would allow for inclusion of children with lower language and cognitive abilities who remain underrepresented in studies of sensory perception in ASD. Finally, we are unable to determine whether the reduced contour integration noted in this study is specific to ASD or characteristic of other developmental disabilities (e.g., ADHD), which are frequently comorbid with ASD. Additional studies to compare across developmental disability populations and to directly assess the role of attention by comparing contour integration in attended versus passive processing would be highly interesting.

In conclusion, children with ASD demonstrate attenuated early automatic neural responses to IC stimuli compared with children with NT development, suggesting reduced deployment of higher-order feedback mechanisms during visual processing of global stimulus features in ASD.
